# Recent advances in treatment for colorectal liver metastasis

**DOI:** 10.1002/ags3.12071

**Published:** 2018-04-17

**Authors:** Eiji Oki, Koji Ando, Ryota Nakanishi, Masahiko Sugiyama, Yuichiro Nakashima, Nobuhide Kubo, Kensuke Kudou, Hiroshi Saeki, Tadahiro Nozoe, Yasunori Emi, Yoshihiko Maehara

**Affiliations:** ^1^ Department of Surgery and Science Graduate School of Medical Sciences Kyushu University Fukuoka Japan

**Keywords:** chemotherapy, colorectal cancer, conversion therapy, liver metastasis, RAS

## Abstract

A major challenge for the management of colorectal liver metastasis (CRLM) is the multidisciplinary approach including surgery. Resection is the most important treatment strategy to prolong the survival of patients with colorectal cancer (CRC). Even when resection is not possible as a primary treatment, it may still be carried out for curative intent after effective chemotherapy. Therefore, resection should always be considered when conducting chemotherapy for CRLM. Neoadjuvant anti‐epidermal growth factor receptor (EGFR) antibody has shown a high response rate for RAS wild CRC. However, whether anti‐EGFR antibody is superior to antivascular endothelial growth factor antibody for all types of CRLM is yet to be determined. Recently, several randomized control trials of first‐line therapy for advanced CRC have been conducted, and some of them are ongoing. The optimal chemotherapy regimen and tumor biology indicated for neoadjuvant chemotherapy as well as conversion surgery are expected to be determined in the near future.

## Abstract 2

Herein, we review and summarize the treatment options for colorectal liver metastasis. We also discuss recent advances in biomarker research for treatment decisions for liver metastasis.

## 1 INTRODUCTION

Colorectal cancer (CRC) is the third most common cancer and the second leading cause of cancer death worldwide.^1^ In Japan, the morbidity and mortality rates of CRC are increasing. According to the 2016 cancer statistics published by the Foundation for Promotion of Cancer Research, more women die from CRC than from any other malignant neoplasm, and it is the third most common cause of cancer death among men, after lung cancer and gastric cancer in Japan.^2^

The 5‐year survival rate for curatively resectable stages I to III CRC is almost 80%, but the 5‐year survival rate for stage IV CRC, which accounts for approximately 18% of cases, is an unsatisfactory 13%. Liver metastases develop in almost 60% of patients with stage IV CRC. Meanwhile, liver recurrence occurs in 9% to 13% of cases after curative resection of CRC. To improve the prognosis of patients with CRC, the therapeutic outcomes for liver metastasis need to be improved. Treatment options for colorectal liver metastasis (CRLM) include liver resection, coagulation therapy, hepatic arterial infusion chemotherapy, and systemic chemotherapy. Of these, liver resection is the most definitive therapy for cure, with 5‐year survival rates of 29% to 48%.^3^, ^4^

Japanese Society for Cancer of the Colon and Rectum (JSCCR) guidelines^5^ recommend curative liver resection in cases in which the liver can be resected without leaving residual metastases, if the primary tumor is controlled or can be controlled, if there are no extrahepatic metastases or they can be controlled, and if remnant liver function can be preserved after resection. Meanwhile, the National Comprehensive Cancer Network (NCCN) guidelines stipulate that the treatment options for liver or lung‐limited synchronous metastases depend on their resectability.^6^ Similar guidelines are applicable for metachronous cancers. However, if the metastases are assessed as resectable, surgery is carried out, whereas systemic chemotherapy followed by evaluations for resectability every 2 months is recommended for unresectable liver metastases. Even cases with multiple metastases can now be cured after resection as a result of recent advances in operative methods and chemotherapy.^7^, ^8^, ^9^

Herein, we review and summarize the treatment options for CRLM. We also discuss recent advances in biomarker research for treatment decisions for liver metastasis.

## 2 CATEGORIES AND GUIDELINES FOR COLORECTAL LIVER METASTASIS

Japanese Society for Cancer of the Colon and Rectum has proposed a classification scheme for CRLM that combines findings of the presence or absence of liver metastases and number and size of metastases. Analysis of registry cases using this classification scheme shows that the proportion of patients undergoing liver resection in categories H2 and H3 are smaller and that the prognoses are poorer than those in H1.^10^ According to the JSCCR guidelines for the treatment of CRC, curative liver resection is recommended if the liver can be resected without leaving residual metastases, if the primary tumor is controlled or can be controlled, if there are no extrahepatic metastases or they can be controlled, and if remnant liver function can be preserved after resection.^11^

NCCN guidelines^12^ indicate that the treatment options for liver or lung‐limited synchronous metastases depend on their resectability. Similar guidelines are applicable for metachronous cancers. The European Society for Medical Oncology guidelines^13^ propose that treatment selection should be guided by the treatment intensity deemed necessary in advanced or recurrent CRC. In cases of wild‐type liver‐only disease, two‐drug combination chemotherapy plus bevacizumab (Bmab) or cetuximab (Cmab) is recommended. Moreover, if the metastases are found to be resectable, resection is advised.

In Europe, Nordlinger et al^14^ proposed treatment guidelines in the European Colorectal Metastases Treatment Group. Disease is categorized as resectable, not optimally resectable, or unresectable. Not optimally resectable is defined as “difficult to resect for technical reasons (proximity to hepatic vein and portal vein branches)” or “technically possible to resect, but oncologically problematic (number of liver metastases greater than four, maximum diameter 5 cm or more, synchronous liver metastases, primary lymph node metastasis positive, and high levels of tumor markers).” Chemotherapy in combination with molecular targeted drugs is recommended, followed by curative resection if a response is achieved.

## 3 LIVER RESECTION FOR CRLM

No standard rules for liver resection after chemotherapy have been established. If the metastasis is technically resectable, then liver resection is usually carried out regardless of the number of tumors. Patient treatment varies substantially among institutions. However, many observational studies reported that resection of liver metastases yielded good long‐term prognosis.^15^, ^16^, ^17^, ^18^

The Japanese Society of Hepato‐Biliary‐Pancreatic Surgery assessed perioperative factors in 727 hepatectomized patients with CRLM between 2000 and 2004 at 11 institutions. They reported 3‐, 5‐, and 10‐year disease‐free survival (DFS) and overall survival (OS) rates of 31.2% and 63.8%, 27.2% and 47.7%, and 24.7% and 38.5%, respectively. They created a nomogram that included six preoperative factors such as synchronous metastases, primary lymph node metastasis, number of tumors, extrahepatic metastasis at hepatectomy, and preoperative tumor marker level to predict the prognosis of patients with CRLM.^19^ Meanwhile, Adam et al^3^ reported that the 5‐year survival rate was higher at 33% in patients who underwent chemotherapy followed by liver resection after an initial assessment of unresectable metastases than in patients who did not undergo liver resection at near 0%. In a clinical study in patients with either liver‐only metastases from CRC or advanced or recurrent CRC, Folprecht et al^4^ found that response rate was strongly associated with resection rate for metastases (liver‐only metastases from CRC, *P* = .002; advanced or recurrent CRC, *P* < .001). However, this does not include clinical studies of concomitant treatment with molecular targeted drugs. Additionally, Kopetz et al^20^ reported the introduction of oxaliplatin in 1998 and the emergence of molecular targeted drugs in 2004 have contributed substantially to improved liver resection rate and thus led to improved OS in advanced and recurrent CRC. By contrast, the LiverMetSurvey study reported that molecular targeted drugs did not contribute to improved OS after curative liver resection.^21^

Recent clinical trials have also shown the contribution of surgery in terms of OS. CALGB/SWOG80405 was a phase III trial of FOLFIRI or mFOLFOX6 with bevacizumab or cetuximab for patients with KRAS wild‐type untreated metastatic adenocarcinoma of the colon or rectum.^22^ A total of 180 cases that were converted to surgery included liver‐only metastasis, and the median survival time of the Cmab arm and Bmab arm was 64.1 months and 67.1 months, respectively. This result showed that 50% of patients survived more than 5 years after conversion surgery. Thus, conversion surgery is considered to contribute to prolonged survival in this patient population.

To improve the resection rate of liver metastasis, portal vein thrombosis, two‐step surgery, and associated liver partition and portal vein ligation for staged hepatectomy (ALPPS) have been conducted.^23^ ALPPS is a unique two‐step hepatectomy technique for obtaining adequate but short‐term parenchymal hypertrophy in oncological patients requiring extended right hepatic resection with limited functional reserve.^24^ ALPPS improves the resectability of CRLM compared with conventional two‐stage hepatectomy. However, ALPPS is still being developed and is associated with high morbidity and mortality. Moreover, the oncological long‐term outcome remains ambiguous. Another study showed that stimulation of liver hypertrophy could accelerate tumor progression.^25^ An observational study on ALPPS^26^ aimed to compare the outcomes of ALPPS in patients with otherwise unresectable colorectal liver metastases and in matched historical controls treated with palliative systemic treatment. In this analysis, unresectable patients who required ALPPS were determined according to at least two of the following criteria: ≥6 metastases, ≥2 future remnant liver metastases, and ≥6 involved segments excluding segment 1. The authors concluded that ALPPS was not superior to systemic treatment with palliative intent. One reason for this result is that the short interval (median 11 days) between stages 1 and 2 of ALPPS does not allow sufficient time for detection of disease progression. Therefore, ALPPS can be used in limited cases only, and two‐stage hepatectomy remains the standard treatment option for multiple liver metastasis.

## 4 ABLATION THERAPY FOR CRLM

Although surgical resection is considered the gold standard for treatment of CRLM, only 10% to 20% of patients with liver metastases are deemed resectable. Metastasis is sometimes contraindicated for surgery because of anatomical reasons. Moreover, patients sometimes have comorbidities or liver dysfunction. In these cases, the patient is ineligible for major surgery. Instead, radiofrequency ablation (RFA) is often applied; however, the role of RFA in the management of CRLM is yet to be elucidated. The CLOCC trial randomized 119 patients with unresectable CRLM between RFA with FOLFOX (±bevacizumab) vs FOLFOX (±bevacizumab) alone.^27^ The authors reported the superiority of RFA with FOLFOX (HR = 0.58, 95% CI: 0.38‐0.88, *P* = .01). Other recent observational studies also showed the superiority of RFA combined with chemotherapy or surgery in terms of prognosis.^27^, ^28^, ^29^ However, some studies have reported the risk of dissemination or incomplete ablation of RFA; therefore, the role of RFA for CRLM is controversial. In general, RFA should be considered in patients who are ineligible for resection as a result of anatomically unresectable lesions, functional insufficiency of hepatic reserve, medical comorbidities, and extrahepatic metastases.

## 5 FOLFOX OR FOLFIRI FOR CRLM

According to previous clinical studies, the resection rate for liver metastasis is higher than that in other sites (Table 1). Selection of chemotherapy regimen is important for CRLM because some unresectable cases can become resectable after chemotherapy. However, the optimal chemotherapy regimen for CRLM is yet to be determined. Whether FOLFOX is better than FOLFIRI or vice versa as a baseline regimen remains unclear. A study conducted by Tournigand et al^30^to compare the usefulness of FOLFIRI vs FOLFIRI followed by FOLFOX as second‐line therapy in advanced or recurrent CRC after first‐line FOLFOX chemotherapy showed that the rate of liver resection in the FOLFIRI‐first group was 9% vs 22% in the FOLFOX‐first group (*P* = .02). Therefore, FOLFOX is often preferred for CRLM. Neoadjuvant chemotherapy is sometimes associated with pathological changes of the liver parenchyma, leading to concerns about toxicity to the remnant liver. There are two types of liver injury: the first involves vascular changes caused by oxaliplatin‐based chemotherapy (sinusoidal dilatation with engorgement of red blood cells associated with sinusoidal obstruction syndrome such as that seen in perisinusoidal fibrosis or venous obstruction),^31^ and the other is steatohepatitis (with severe steatosis, lobular inflammation, or hepatocyte ballooning) caused by irinotecan‐based chemotherapy. Steatohepatitis as a result of irinotecan‐based chemotherapy possibly increasing the 90‐day mortality rate (14.7%) is a cause for concern.^32^

**Table 1:** Results of liver resection in clinical studies for advanced or recurrent colorectal cancer

Study title	No. of patients	ORR (%)	Resection rate (%)	R0 resection rate (%)	Liver resection rate^a^ (%)	R0 liver resection rate^a^ (%)
First‐BEAT^37^						
Oxaliplatin‐based CT + Bmab	949	NR	16.1^b^	12.2^b^	20.3	15.4
Irinotecan‐based CT + Bmab	662	NR	9.7	7.4	14.3	11.7
NO16966^38^						
FOLFOX/XELOX	701	38	6.1^b^	4.9^b^	NR	11.6
FOLFOX/XELOX + Bmab	699	38	8.4	6.3	NR	12.3
CRYSTAL^39^						
FOLFIRI (RAS wild)	599	38.7 (38.6)	3.7^b^	1.7^b^	NR	4.3
FOLFIRI + Cmab (RAS wild)	599	46.9 (66.3)	7.0	4.8	NR	9.8
OPUS^40^						
FOLFOX (RAS wild)	168	36 (29)	NR	2.4^b^	3.6^b^	NR
FOLFOX + Cmab (RAS wild)	169	46 (58)	NR	4.7	6.5	NR
Fire‐3^71^						
FOLFIRI + Bmab (RAS wild)	295	58 (60)	14^b^	NR	NR	NR
FOLFIRI + Cmab (RAS wild)	297	62 (65)	12	NR	NR	NR
TRIBE^67^						
FOLFIRI + Bmab (RAS wild)	256	54 (56)	NR	12^b^	NR	NR
FOLFOXIRI + Bmab (RAS wild)	252	65 (63)	NR	15	NR	NR
WJOG4407G^44^						
FOLFOX + Bmab	198	62	13^b^	9^b^	NR	NR
FOLFIRI + Bmab	197	64	12	10	NR	NR
SOFT^45^						
FOLFOX + Bmab	255	62.7	NR	9^b^	NR	NR
SOX + Bmab	256	61.5	NR	9	NR	NR

a Proportion of patients with liver metastases.

b % of total no. of patients.

CT, chemotherapy; NR, not reported; ORR, overall response rate.

In combination with chemotherapy, the antiangiogenic drug bevacizumab protects against pathological changes of the liver parenchyma caused by chemotherapy, and its pathological benefits suggest that it could potentially improve prognosis.

In a retrospective study of sinusoidal dilatation in 105 patients who underwent liver resection after fluorouracil (5‐FU)/oxaliplatin therapy with or without concomitant bevacizumab, Ribero et al^33^ showed that the incidence of Rubbia‐Brandt Grade 2‐3 sinusoidal dilatation was 27.9% in patients treated without bevacizumab versus 8.1% in patients treated with bevacizumab (*P* = .006). Recently, in addition to inhibiting the development of oxaliplatin‐induced sinusoidal dilatation, bevacizumab has also been reported to potentially inhibit splenomegaly and thrombocytopenia by inhibiting portal hypertension.^34^

## 6 NEOADJUVANT CHEMOTHERAPY FOR RESECTABLE CRLM

Usefulness of neoadjuvant chemotherapy (perioperative chemotherapy) for patients with CRC with up to four liver metastases was verified in the EORTC 40983 study.^35^ In total, 364 patients were randomly assigned to treatment with either six cycles of pre‐ and postoperative FOLFOX4 (n = 182) or resection only (n = 182). In the intention‐to‐treat (ITT) analysis, the 3‐year progression‐free survival (PFS) rates in the perioperative chemotherapy group and in the resection‐only group were not significantly different at 35.4% and 28.1%, respectively (HR = 0.79; *P* = .058). However, in eligible patients, the 3‐year PFS rates were 36.2% and 28.1% (HR = 0.77; *P* = .041) and 42.4% and 33.2% (HR = 0.73; *P* = .025) in the chemotherapy and resection‐only groups, respectively, indicating that prognosis was significantly better in the perioperative chemotherapy group. The new EPOC trial aimed to assess the benefit of adding cetuximab to standard chemotherapy in patients with resectable colorectal liver metastasis.^36^ A total of 257 patients with KRAS exon 2 wild‐type resectable or suboptimally resectable colorectal liver metastases were randomized in a 1:1 ratio to receive chemotherapy with or without cetuximab before and after liver resection. PFS was significantly shorter in the chemotherapy plus cetuximab group than in the chemotherapy alone group (14.1 months [95% CI: 11.8‐15.9] vs 20.5 months [95% CI: 16.8‐26.7], HR = 1.48, 95% CI: 1.04‐2.12, *P* = .030). This result indicates that the addition of cetuximab to neoadjuvant chemotherapy for operable colorectal liver metastases is not recommended. The usefulness of neoadjuvant chemotherapy for patients with resectable CRLM is still under debate.

## 7 CHEMOTHERAPY FOR UNRESECTABLE CRLM

Several clinical studies have found that the liver became resectable during subsequent chemotherapy in patients who were initially deemed to have unresectable disease.^37^, ^38^, ^39^, ^40^, ^41^, ^42^, ^43^ These studies are categorized into two types: those that target unresectable CRC or unresectable colorectal liver‐only metastasis.

In the clinical studies that target unresectable CRC, the resection rate of liver metastasis is 4% to 15%^38^, ^39^, ^40^, ^44^, ^45^ (Table 1). However, in these trials, the patient with liver metastasis was not an allocation factor; thus, exact evaluation of the effect of chemotherapy for liver metastasis is difficult.

In clinical studies that targeted liver‐only metastasis (Table 2), the anti‐epidermal growth factor receptor (EGFR) antibody cetuximab excellently improved the response rate and yield of curative liver resection, for which it has attracted attention. However, the definitions of unresectable liver metastasis in each clinical study for liver‐only metastases from CRC varied among these studies (Table 3). Folprecht et al^46^ reported the results of a randomized phase II study of FOLFOX/FOLFIRI plus cetuximab in patients with liver‐only metastases from CRC. Among the 106 patients evaluated, the response and R0 resection rates were 68% and 38%, respectively, in the 53 patients receiving FOLFOX plus cetuximab, whereas they were 70% and 33%, respectively, in the 67 patients with KRAS wild‐type status.

**Table 2:** Results of liver resection in clinical studies for liver metastases from colorectal cancer

Study title	No. of patients	Response rate (%)	Liver resection rate (%)	R0 liver resection rate (%)
Alberts et al^41^				
FOLFOX	42	60	40	33
BOXER^42^				
XELOX + Bmab	45	78	36	20
CELIM^46^				
FOLFOX + Cmab	53	68	51	38
FOLFIRI + Cmab	53	57	49	30
POCHER^43^				
FOLFOXIRI + Cmab	43	79	60	60
OLIVIA^47^				
FOLFOX + Bmab	39	62	NR	23
FOLFOXIRI + Bmab	41	81	NR	49
PLANET^48^				
FOLFOX+Pmab (RAS wild)	38	74 (78)*	45	34 (R0+R1)
FOLFIRI+Pmab (RAS wild)	39	67 (73)*	59	46 (R0+R1)

NR, not reported.

*RAS wild only.

**Table 3:** Definitions of curatively unresectable in clinical studies

Study	Definition
Alberts, et al.	Multiple liver metastases in both hepatic lobes Proximity of tumor to major vascular structures, preventing preservation of an adequate hepatic remnant Large tumor jeopardizing remnant liver function
BOXER	Five or more liver metastases Liver metastasis diameter larger than 5 cm Location and distribution of metastatic disease within the liver unsuitable for resection Residual liver parenchyma volume not adequate for maintaining viable liver function Unable to retain adequate vascular flow to maintain viable liver function Synchronous liver metastases
CELIM	Five or more liver metastases For technical reasons, is concluded to be unresectable or difficult‐to‐resect Judged to be technically unresectable, in light of remaining hepatic function Invasion into all hepatic veins is evident Invasion into both right and left hepatic arteries or portal veins is evident
POCHER	Five or more liver metastases Diameter larger than 5 cm Hilar metastasis, extrahepatic distant metastasis (except micronodular lung metastases)
OLIVIA	No upfront R0/R1 resection of all hepatic lesions possible <30% estimated residual liver volume after resection Metastases in contact with major vessels of the remnant liver

Addition of irinotecan in the 5‐fluorouracil/folinic acid, oxaliplatin, irinotecan (FOLFOXIRI) regimen is effective for tumor shrinkage in CRLM.^47^ The OLIVIA trial assessed the efficacy of bevacizumab plus modified FOLFOX‐6 or FOLFOXIRI for patients with initially unresectable CRLM. Overall tumor response rate of the FOLFOXIRI arm was 81% (95% CI: 65%‐91%), and the overall resection rate was 61% (95% CI: 45%‐76%).^47^ FOLFOXIRI + BV is also effective for patients with BRAF mutation, and this regimen can be selected for conversion therapy.

Regarding anti‐EGFR antibody, panitumumab is effective for increasing the overall response rate (ORR) and resection rate.^48^, ^49^ However, when determining the optimal multidisciplinary treatment strategy for KRAS wild‐type liver‐limited, initially unresectable CRC, no unequivocal evidence shows that molecular targeted therapy in combination with chemotherapy is better with either anti‐VEGF antibody or anti‐EGFR antibody despite the presence of data suggesting that “liver resection rate,” “improvement of response rate,” and “pathological improvement” improve prognosis. Recently, it was reported that anti‐EGFR antibodies do not have survival benefit for RAS wild‐type right‐side colon cancer.^50^, ^51^ However, the shrinkage of tumor is adequate even in right‐side colon cancer.^52^, ^53^ Therefore, anti‐EGFR antibodies should be used cautiously for right‐side RAS wild‐type CRLM.

Previously, we conducted two independent phase II trials that targeted patients with CRLM, namely, KSCC0802 and KSCC1002.^54^, ^55^ In the KSCC0802 multicenter trial, 40 patients with unresectable CRLM were included and received mFOLFOX6 + Bmab. Meanwhile, 33 patients with KRAS wild‐type with unresectable CRLM were included from a trial of SOX (S‐1 and oxaliplatin) plus Cmab in the KSCC1002 trial. In the KSCC0802 trial (mFOLFOX6 plus bevacizumab), the ORR was 42.5%, and R0 resection was achieved in 25% of the enrolled patients after chemotherapy. In the KSCC1002 trial (SOX plus cetuximab), the ORR was 63.6%, and R0 resection was achieved in 39.4% of the enrolled patients after chemotherapy. High tumor shrinkage mediated by SOX plus cetuximab led to high resectability for CRLM. Therefore, in the ATOM study, we planned a randomized phase II clinical study to conduct an exploratory comparison of mFOLFOX6 plus bevacizumab versus mFOLFOX6 plus cetuximab in KRAS wild‐type, difficult‐to‐resect, liver‐only metastases from CRC. We also investigated the differences in pathological response and morphological response between Bmab and Cmab. The results will be published in 2018.

## 8 ADJUVANT CHEMOTHERAPY AFTER RESECTION OF CRLM

In an investigation of the role of adjuvant chemotherapy following liver resection, the FFCD ACHBTH AURC 9002 study compared two treatments after curative liver resection: 5‐FU/leucovorin (LV) for 6 months (n = 86) versus surgery alone (n = 85).^56^ The 5‐year disease‐free survival rates in the 5‐FU/LV group and in the surgery‐only group were 33.5% and 26.1% (odds ratio [OR] = 0.66; *P* = .028), and the 5‐year OS rates were 51.1% and 41.1% (OR = 0.73; *P* = .13), respectively. A pooled analysis of the results of the French FFCD study and the English ENG study also showed that adjuvant 5‐FU/LV is potentially more useful than resection alone (median PFS was 27.1 months vs 18.8 months, respectively; HR = 1.32; *P* = .058).^57^

In Japan, Hasegawa et al^58^ reported that adjuvant therapy with uracil‐tegafur and leucovorin (UFT/LV) effectively prolongs recurrence‐free survival (RFS) after hepatic resection for CRLM and can be recommended as an alternative treatment modality. The JCOG0603 study, which aims to compare FOLFOX with resection only after curative resection, is currently underway.^59^ Collectively, these results indicate that 5‐FU monotherapy is effective for adjuvant chemotherapy after surgery of CRLM.

## 9 BIOMARKERS FOR CRLM

Impact of tumor biology on prognosis in patients with CRLM has been the topic of intense research. Some systematic literature reviews show that KRAS and BRAF V600E mutations are negatively associated with OS and RFS in patients who undergo complete liver resection for CRLM.^60^, ^61^, ^62^, ^63^ In particular, BRAF V600E mutations that present in 8%‐10% of patients are consistently associated with poor prognosis and result in possible patient ineligibility for resection of CRLM.^64^, ^65^

Recently, a small single‐center cohort study showed that 21 of 52 patients with BRAF V600E mutant who underwent metastasectomy had longer OS (29.1 months vs 22.7 months) and PFS (13.6 months vs 6.2 months) compared with the non‐metastasectomy cohort. The authors concluded that multimodality therapy incorporating metastasectomy for BRAF V600E metastatic CRC (mCRC) should be considered and might be associated with improved OS in selected patients.^66^ Meanwhile, BRAF V600E can be a biomarker for selecting the appropriate chemotherapy regimen. Currently, FOLFOXIRI + BV might be the only effective regimen for multimodality treatment of the patient with BRAF V600E mutation;^67^ therefore, BRAF mutation analysis should be done before treatment of CRLM.

Microsatellite instability (MSI) status or mismatch repair deficiency (MMR‐D) has been the biomarker for adjuvant 5‐FU monotherapy and immune checkpoint inhibitor. Hematogenous and lymphogenous metastasis‐dominant CRC with high‐frequency MSI (MSI‐H) are reported to have poor prognosis.^68^ However, the validity as the prognostic factor of MMR is yet to be confirmed, and it should thus be used cautiously. Primary location of the tumor is also a factor in treatment decision. Recent studies reported that right‐sided primary tumors might be more likely to recur.^69^, ^70^ In particular, palliative resection might not be done because these patients showed no benefit from resection.^69^

Tumor biology should be further studied for precise treatment of CRLM.

## 10 CRLM TREATMENT

**Figure 1:**
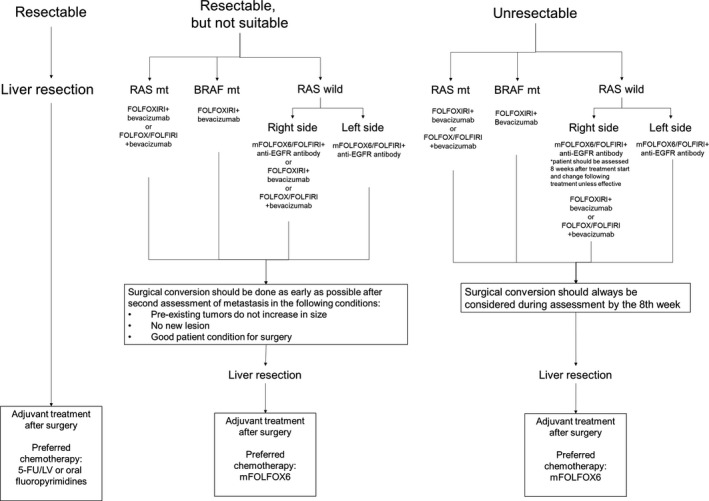
Treatment flow for colorectal cancer liver metastasis. EGFR, epidermal growth factor receptor; 5‐FU/LV, fluorouracil/leucovorin

In the process of liver resection, the liver surgeon first determines whether or not a liver metastasis is resectable; one to three liver metastases within 5 cm can be resectable, but the judgment of the liver surgeon is necessary because factors such as location of the liver metastasis, liver function, and patient condition should be considered carefully Figure 1 shows CRLM treatment.

Liver metastases are classified into three types as follows: resectable, resectable but not suitable; and unresectable. The resectable cases should undergo resection first but chemotherapy should be given after resection. The resectable but not suitable cases and the unresectable cases should be given chemotherapy, but the choice of chemotherapy depends on tumor location and RAS and BRAF mutation status. In the case of RAS mutant, FOLFOXIRI + BV is highly recommended, but FOLFOX + BV is another choice. In the case of BRAF mutant, FOLFOXIRI + BV is strongly recommended. In the case of RAS wild and right‐side CRLM, FOLFOX + anti‐EGFR antibody is recommended in terms of response rate, but FOLFOX/FOLFIRI + anti‐EGFR antibody should not be used for long periods without confirmation of tumor response. If tumor reduction is less than 30% 8 weeks after the start of treatment, the regimen should be changed to FOLFOXIRI + Bmab or FOLFOX/FOLFIRI + Bmab.

In the case of RAS wild and left‐side CRLM, FOLFOX and anti‐EGFR antibody are recommended.

In all cases, the liver metastasis should be re‐evaluated at the eighth week of treatment. Resection should always be considered, and the chance for resection should not be overlooked, particularly in the resectable but not suitable cases.

## 11 CONCLUSION

Because many agents have been developed in this decade, a proportion of CRLM cases have changed to curative disease. Patients with CRLM are considered to have stage IV disease, but they are always potential candidates for curative resection, if the metastases are limited within the liver. Therefore, the goal of chemotherapy is conversion surgery for curative intent. In these situations, optimal multimodality treatment option that includes chemotherapy, surgery, and radiology is essential.

## DISCLOSURE

Conflicts of interest: Y.M. received research grants from Yakult Honsha, Merck Serono, Chugai Pharma, Takeda Pharma, and Taiho Pharma; E.O. received lecture fees from Yakult Honsha, Merck Serono, Chugai Pharma, Takeda Pharma, and Taiho Pharma. The other authors have no conflicts of interest to declare.
